# Stabilization of perturbed Boolean network attractors through compensatory interactions

**DOI:** 10.1186/1752-0509-8-53

**Published:** 2014-05-08

**Authors:** Colin Campbell, Réka Albert

**Affiliations:** 1 Department of Physics, The Pennsylvania State University, University Park, PA 16802, USA; 2 Department of Biology, The Pennsylvania State University, University Park, PA 16802, USA; 3 Pennsylvania State University, 208 Mueller Laboratory, University Park, PA 16802, USA; 4 Pennsylvania State University, 152E Davey Laboratory, University Park, PA 16802, USA

**Keywords:** Boolean networks, Discrete dynamic models, Signal transduction, Stability, Attractors, Network manipulation, Interaction modification, T-LGL leukemia, Abscisic acid signaling

## Abstract

**Background:**

Understanding and ameliorating the effects of network damage are of significant interest, due in part to the variety of applications in which network damage is relevant. For example, the effects of genetic mutations can cascade through within-cell signaling and regulatory networks and alter the behavior of cells, possibly leading to a wide variety of diseases. The typical approach to mitigating network perturbations is to consider the compensatory activation or deactivation of system components. Here, we propose a complementary approach wherein interactions are instead modified to alter key regulatory functions and prevent the network damage from triggering a deregulatory cascade.

**Results:**

We implement this approach in a Boolean dynamic framework, which has been shown to effectively model the behavior of biological regulatory and signaling networks. We show that the method can stabilize any single state (e.g., fixed point attractors or time-averaged representations of multi-state attractors) to be an attractor of the repaired network. We show that the approach is minimalistic in that few modifications are required to provide stability to a chosen attractor and specific in that interventions do not have undesired effects on the attractor. We apply the approach to random Boolean networks, and further show that the method can in some cases successfully repair synchronous limit cycles. We also apply the methodology to case studies from drought-induced signaling in plants and T-LGL leukemia and find that it is successful in both stabilizing desired behavior and in eliminating undesired outcomes. Code is made freely available through the software package BooleanNet.

**Conclusions:**

The methodology introduced in this report offers a complementary way to manipulating node expression levels. A comprehensive approach to evaluating network manipulation should take an "all of the above" perspective; we anticipate that theoretical studies of interaction modification, coupled with empirical advances, will ultimately provide researchers with greater flexibility in influencing system behavior.

## Background

Characterizing the deregulation of signaling networks is a crucial component of understanding a variety of diseases, including diabetes [1,2] developmental disorders [3], and cancer [3-5]. For instance, cellular signaling networks may be damaged via mutations or changes in the activation levels of constituent components, which may lead to abnormal cell behavior [6]. A specific example is the mutation of the transforming growth factor (TGFβ) receptor protein that leads to unregulated cell growth [7].

Signaling networks are one class of complex systems: i.e., collections of interacting components that, on a holistic level, display behavior that cannot be predicted from analysis of the system's components in isolation. Complex systems exist in diverse biological, social, and physical contexts. Network science has developed as an effective framework with which to study complex systems. Biologists use network representations of intra- and inter- cellular signaling to study diverse phenomena, including pathogen-immune system interactions [8,9], cancer progression [10-12], and regulatory behavior in the face of changing environmental conditions [13,14]. On a larger biological scale, networks have been used to model ecological interactions, including predator–prey food webs [15] and mutualistic interactions between, for instance, plants and their pollinators [16]. Networks have also been used to effectively model human social interactions, especially in the context of the spread of disease [17], opinions [18], and information [19] through a population. Network theory has elucidated the emergent properties of technological systems, including the World Wide Web and Internet, and is used to model power grids and roads [17].

A **network**, in its most basic form, consists of nodes (denoting system components) and edges between nodes (representing interactions and relationships among components). The structure of a network, quantified by network measures such as the degree distribution, clustering coefficient or distance [17,20], can be information-rich. For example, the topology of a social friendship network can be used to determine friendship cliques and key social mediators [21,22]. However, the topology alone frequently provides an incomplete view of the system. The propagation of a signal through an intra-cellular network, for instance, must be represented by assigning a dynamic activation level to each node of the network and quantifying the regulatory relationships between nodes.

**Figure 1:**
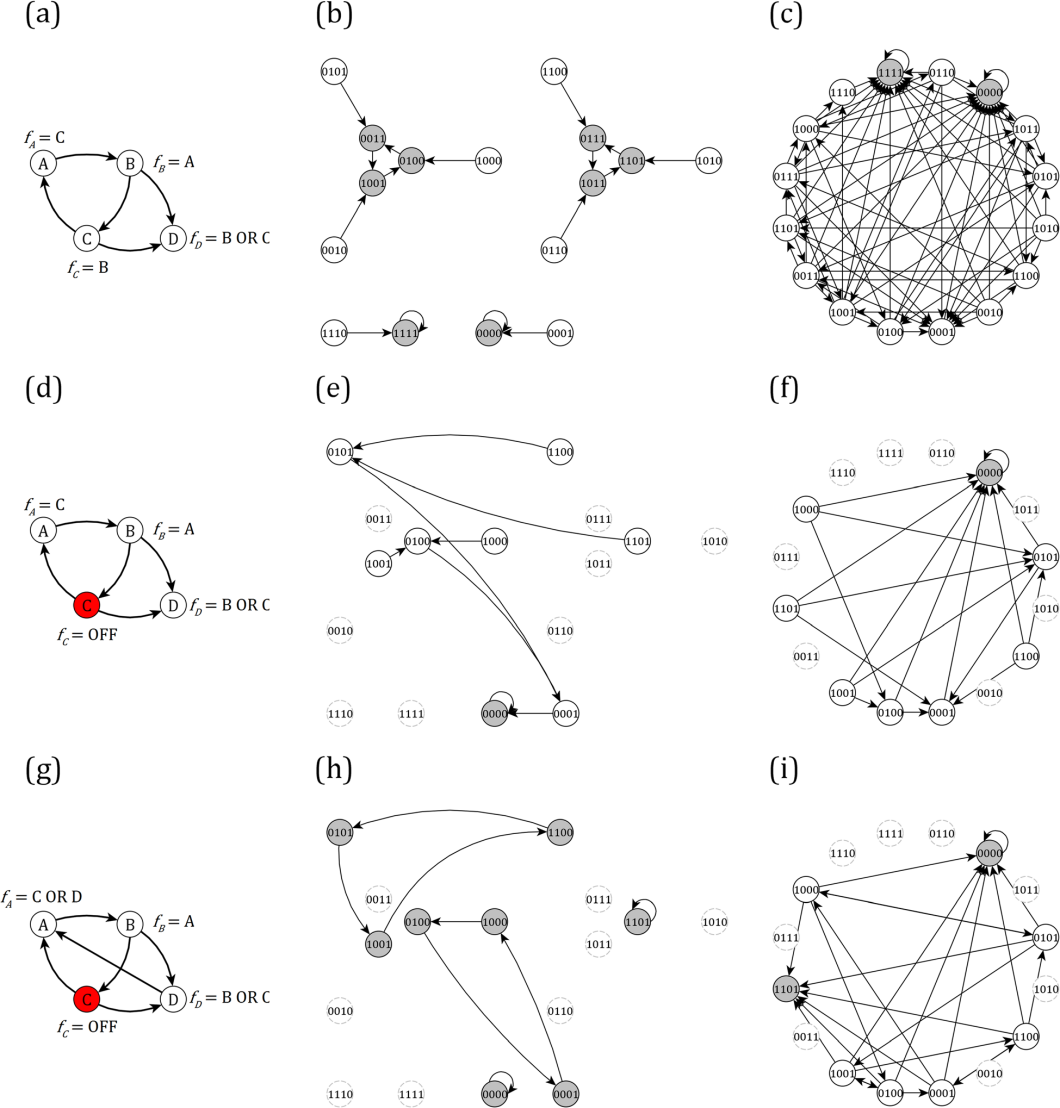
**Illustration of network damage and the methodology to repair a steady state. (a)** A four-node network with logical update rules. The corresponding state transition networks under synchronous and random order asynchronous dynamics are shown in panels **(b)** and **(c)**, respectively. Node labels indicate the state of each node in alphabetic order. Panels **(d-f)** show the same information for the network damaged such that node C is always OFF (0). The network states where node C is ON (1) are shown in panels **(e-f)** for completeness, but because they are no longer accessible to the network, they are shown in faded gray. Panels **(g-i)** show one repair methodology that ensures that the state 1101, where all nodes aside from node C are ON (1), is a steady state of the network.

The Boolean framework has become a standard methodology for modeling dynamical processes on networks, especially in biological contexts [23-25] (Figure 1(a)). In a **Boolean model of a network**, nodes are assumed to be either active or inactive (i.e., ON or OFF, or equivalently 1 or 0). The nodes are inter-related such that the dynamics of an arbitrary node *x*_*i*_ obeys *x*_*i*_(*t* + *τ*_*i*_) = *f*_*i*_(*x*_1_(*t*), …, *x*_*N*_(*t*)), where for simplicity each node’s state (ON or OFF) is denoted by the node name *x*_*i*_ and *τ*_*i*_ is the time delay (response time) of node *x*_*i*_. Depending on the system being modeled, the regulatory relationships (the *f*_*i*_ functions) may be represented by logical functions [24,26], threshold rules [27,28], or truth tables [29,30], which give the next state of a regulated node for every combination of its regulators’ current states. For example, the logical function *f*_*A*_ *= (B AND C) OR D* indicates the relationship between the future state of node A and the current states of its regulators, nodes B, C and D. Specifically, node A will be ON in the future if either B and C are ON simultaneously, or D is ON. The dynamic updating process (recalculation of each node’s state according to its regulatory relationship) used in Boolean models is often done in **discrete time**: node states are recalculated either **synchronously** (simultaneously), wherein *τ*_*i*_ *= 1* for any *i* or **asynchronously,** wherein node-dependent time delays are used or, equivalently, node states are updated in a prescribed or random sequence. Time discretization is clearly an abstraction of the real system being modeled, where interactions occur in continuous time and over differing time scales. In situations where these time scales are not known and therefore cannot be integrated into discrete dynamic network models, as is often the case in biological systems [24], stochastic asynchronous models offer a method of sampling all possible time scales. In this way, these models capture a broad range of possible dynamical behavior; while such predictions are necessarily imprecise, dynamic Boolean models are attractive in that they do not require extensive parameterization (and thereby capture behavior that arises from the fundamental characteristics of the interactions between system components). Indeed, these models have been shown to effectively capture the qualitative behavior of a variety of real systems (see e.g. [23,31-33]).

In a Boolean framework, the state of a network with *N* nodes at any time step may be represented by a Boolean vector of length *N*; each bit represents the state of a node (Figure 1(b)). The procession between the 2^*N*^ states may be mapped in the so-called **state transition network**, whose nodes are the states of the system and whose directed edges indicate state transitions that are allowed by the model. After sufficient applications of the network update rules, the network enters into a state or a closed set of states from which it cannot escape, called an attractor. An attractor can be a **steady state** (a fixed point that the network never leaves), or a **limit cycle** (a set of states through which the system cycles, deterministically for synchronous update schemes or probabilistically for stochastic asynchronous update schemes). The attractors of a network represent the stable dynamical configurations of the system. The states that can reach a specified attractor through a path of successive state transitions form the basin of attraction of the attractor. In the context of a cellular signal transduction network, the attractors may, for instance, represent cell fates such as death or unregulated growth [34]. As such, determining the attractors of a network, and characterizing their behavior in the face of system perturbations (i.e., a transient or permanent change in the state of a node and/or in the interactions between nodes), are major foci of dynamic Boolean network analysis. Significant effort has been devoted to characterizing the effects of damage on a network's attractors (see e.g. [35,36]). While the perturbation of a node can, in some cases, have no effect on any other nodes (in which case a near-identical attractor that only differs in the state of the perturbed node is preserved), often the perturbation leads to a cascading failure and finally the system stabilizes in a different attractor.

Not all system perturbations, however, necessarily represent damage to the system. A natural counterpart to the study of network damage is the study of network control: network manipulations brought about by human intervention to influence the behavior of the network. In general, network control involves driving the state of a network from an initial state to a final target state. For example, Liu et al. have shown that networks with linear dynamics are fully controllable (can be driven from any initial state to any target state) if the state of roughly 80% of the nodes are externally manipulated [37]. One can also envision more specific intervention strategies whose objective is to drive the system from an initial undesirable state (for example, a state caused by network damage) to a more desirable target state (for example, a state as close as possible to the undamaged state). This type of mitigating intervention is expected to involve the control of fewer nodes than full controllability and thus it should be more practical.

While the most obvious mitigating intervention in response to network damage is the direct reversal of the damage, this may be impractical to implement in the system under study (for example, there may not exist a drug to target the deregulated protein). Moreover, the initial damage may have cascading effects that cannot be undone by only reversing the initial damage. For example, the loss of a species in a food web can lead to a catastrophic collapse in the local ecosystem [38], at which point the re-introduction of the originally lost species will likely not suffice for the restoration of the original food web.

An alternative approach to the direct reversal of the initial damage involves fixing the state of one or more nodes that are not part of the original damage (see e.g. [34,39]). These compensatory perturbations aim to move the system to the basin of attraction of a desirable attractor which is as close as possible to the attractor of the undamaged network. Here, we consider a complementary approach, wherein we modify network edges (e.g., regulatory relationships) rather than node states; this approach has received comparatively little attention in dynamical networks (but see [40] for such a study in static networks). Moreover, the control problem we are considering is not of changing the state of the system, but of changing the stability of the state that the system enters as a direct result of the damage.

Our goal is to identify interventions that can be used preemptively to mitigate the cascading effects of network damage. The method of selecting these interventions is based on developing an understanding of the first deviations caused by the damage: in a regulatory chain A - > B - > C wherein node A is damaged, we wish to modify the regulatory relationships such that node B is not deregulated as an effect of the damage. If this goal is accomplished, the stability of node C is assured. For example, by developing an understanding of the first effects of overactive TGFβ signaling, the identified interventions would prevent them from happening and thus prevent unregulated cell growth. An important caveat of this approach is that in an empirical system a deregulatory cascade occurs dynamically in real time; the application of the regulatory intervention must therefore occur immediately after or even before detecting the initial damage.

Specifically, in this article we consider Boolean networks with logical update rules. We express network damage in the form of a sustained ON or OFF state of a node, regardless of the state of the node’s regulators. This type of abnormal state is often encountered as an effect of gene mutations that render the encoded protein not expressed or nonfunctional or, conversely, constitutively expressed or over-active. We develop an algorithm that identifies simple modifications to node interactions that allow the damage-modified attractor to remain an attractor in the damaged network. We apply the algorithm to random Boolean networks with a synchronous update scheme in order to show its general effectiveness and limitations. We then apply the approach to two biological case studies. We first show that the methodology identifies numerous potential interaction modifications to restore abscisic acid signaling in plants in response to the loss of key regulatory components. We then show that the methodology may be readily extended to remove an undesired (cancerous) attractor in a network model of T cell large granular lymphocyte leukemia.

## Methods

### Network properties

The state of an *N* node Boolean network is described by a Boolean vector of length *N*: [*x*_1_(*t*), …, *x*_*N*_(*t*)], where *x*_*i*_(*t*) ∈ {0, 1}. Each node *x*_*i*_ has an update function *x*_*i*_(*t* + *τ*_*i*_) = *f*_*i*_(*x*_1_(*t*), …, *x*_*N*_(*t*)) that determines its dynamics; here we express these update functions as logical update rules. Consider the four-node network shown in Figure 1(a). Under synchronous dynamics, where all nodes are updated at multiples of a common time step, and thus *τ*_*i*_ *= 1* for all *i*, the network has two steady states and two limit cycle attractors (Figure 1(b)). Asynchronous dynamics are commonly generated via the random order asynchronous (ROA) update scheme or the general asynchronous (GA) update scheme. In the ROA method the time delays are randomly selected such that every sequential update of the nodes has an equal chance. In other words, a random permutation of the nodes is generated, the state of each node is updated using the most recent states of its regulators, then a different permutation is selected randomly, and so on. In the GA method, a node is randomly selected to be updated at every time step; unlike in the ROA update scheme, a node in the GA update scheme may be updated twice before every node is updated once. A key observation is that the steady states of a Boolean network are independent of the value of the time delays or of the order of update; i.e., independent of the choice of synchronous, ROA, GA, or other update scheme [24]. In contrast, limit cycle attractors encountered for synchronous update may not be preserved when switching to asynchronous update [24]. We apply the ROA update scheme to the network of Figure 1(a), and show (Figure 1(c)) that it has two steady state attractors that coincide with the steady states under synchronous update (Figure 1(b)).

We consider network damage in the form of node knockout or constitutive expression; i.e., the update function for a damaged node *x*_*i*_ becomes *x*_*i*_(*t* + *τ*_*i*_) = 0 or *x*_*i*_(*t* + *τ*_*i*_) = 1. This alters the dynamical behavior of the nodes regulated by node *x*_*i*_, which leads to a modified state transition network. For example, if node C is knocked out, in both dynamical schemes the network's only attractor becomes the "all OFF" (0000) steady state (Figure 1(d-f)).

We wish to modify the node update rules to restore a specific attractor of the original network, given that the damage may not be directly reversed (e.g., in the example of Figure 1, given that node C will remain fixed in the OFF state). Consider *a*_*s*_, a steady state attractor of the original network. The network damage sends the state of the network from *a*_*s*_ to *a*^*d*^: a state identical to *a*_*s*_ other than for the state of the damaged node. For example, considering the "all ON" (1111) steady state in the example of Figure 1, after knocking node C out the state becomes *a*^*d*^ = 1101; the goal is then to make this state a steady state. The state *a*^*d*^ is not in general a steady state of the damaged network, and so we modify the update rules such that it becomes one, i.e. $a^{d}\to a_{s}^{d}$.

**Figure 2:**
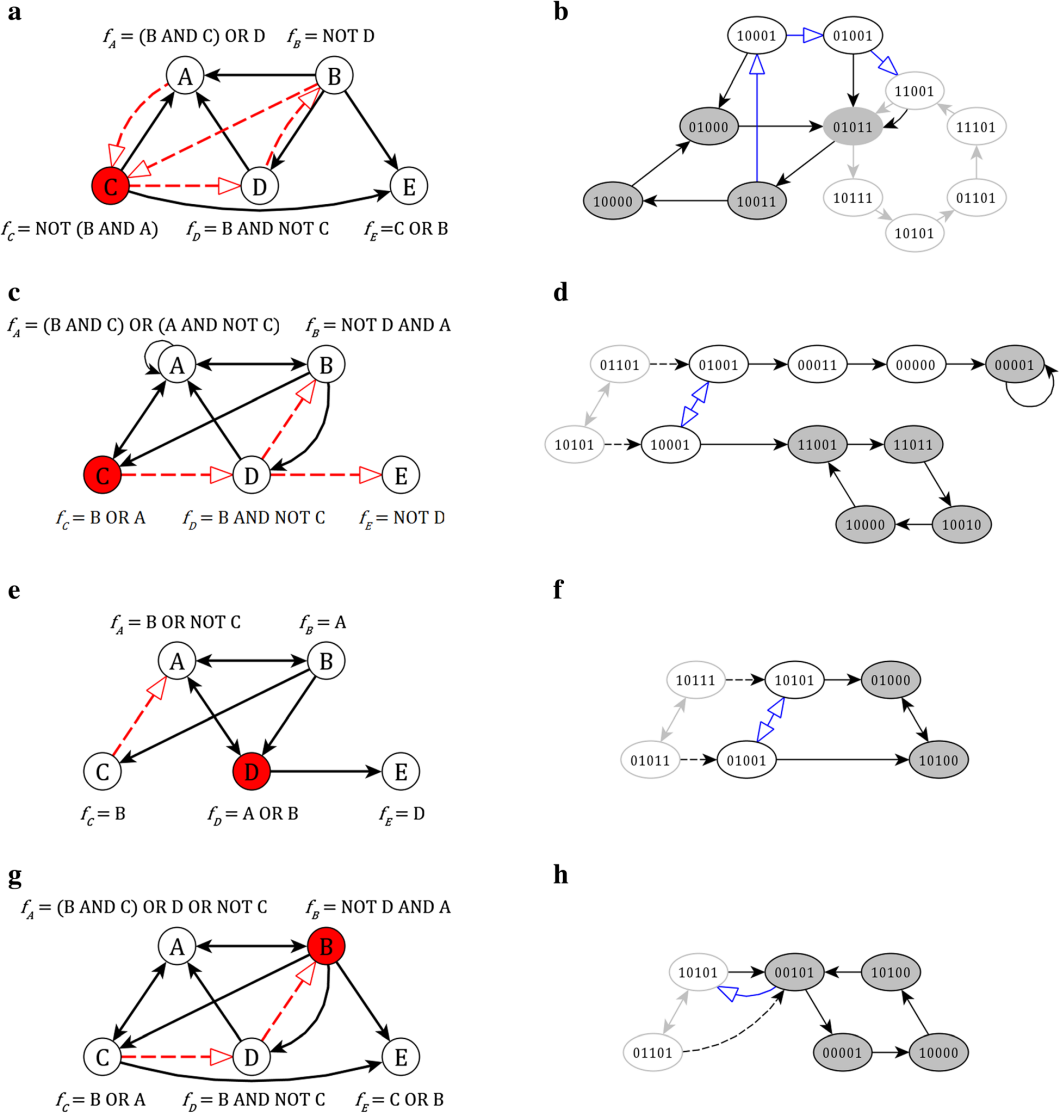
**Illustration of limit cycle repair.** The left column indicates four networks with logical update rules. In each network, a node *x* is marked in red to indicate *f*_*x*_ = OFF when the network is damaged. Black, solid arrows indicate positive or mixed regulation and red, dashed arrows indicate negative regulation, as described in the accompanying Boolean update rules. The right column shows a portion of the synchronous state transition networks corresponding to each network on the left. States with a grey border correspond to a limit cycle of the undamaged network, *a*, with grey arrows indicating the procession among the states. States with a grey fill correspond to an attractor of the damaged network, with solid black arrows indicating the procession among the states. All other states act as transient states in the damaged network. Dashed black arrows indicate the modification of a state due to the damage (i.e. the turning off of the red node). Blue arrows with unfilled arrowheads indicate desired transitions, to be achieved through interaction modification, that stabilize the damage-equivalent attractor *a*^*d*^. **(a-b)** An example of a limit cycle where the network damage reduces the size of the repairable limit cycle (see Methods). Panels **(c-d)**, **(e-f)**, and **(g-h)** show limit cycles that cannot be repaired with the methodology presented in this report, illustrating cases 1, 2, and 3 (see Methods).

Similarly, in the case of a set of limit cycle states *a*_1_, …, *a*_*n*_ (*n* > 1), we wish to ensure that the parallel damaged states $a_{1}^{d},\ldots ,a_{n}^{d}$ constitute a limit cycle of the damaged network. We note that some states in a limit cycle may collapse due to network damage (e.g., states 101 and 001 merge into 101 if the first node is fixed to be ON). In these cases we choose as the target of our mitigation strategy the largest attractor that can be formed from the $a_{1}^{d},\ldots ,a_{n}^{d}$ states. This reduces the length of the attractor but ensures that no ambiguity arises as a result of the reduction in the size of the state space. For instance, the network shown in Figure 2(a) has a six-state synchronous limit cycle (grey-bordered states with gray edges, Figure 2(b)). Knocking out node C results in the formation of a four-state synchronous limit cycle (grey-filled states, Figure 2(b)). When considering the damage-modified version of the attractor states, two are identical to the original attractor (11001 and 01011) and three translate unambiguously to a new state (10111 - > 10011, 10101 - > 10001, and 01101 - > 01001). The sixth state, 11101, translates to a pre-existing state of the attractor, 11001. Thus, the desired repaired limit cycle contains five states, rather than the original six. The blue edges with open tips indicate the transitions that must be enacted through network repair to force this set of five states to be an attractor of the repaired network.

### Form of interaction modifications

We provide an overview of the network repair algorithm in Table 1. However, before discussing it in depth, we first address the general form of the interaction modifications considered in this article (Table 2). We separately consider interaction modifications that involve changing a node from updating to ON to updating to OFF and vice versa (left and right columns of Table 2, respectively). We here restrict ourselves to relatively minor functional modifications, in an effort to generate practically implementable predictions. We consider update functions as logical rules, and choose to scaffold upon the original update function by adding terms, rather than by removing pre-existing terms. In all cases, we add an interaction to the update rule for node *x* with a node that previously played no part in its update function. In some cases, we additionally add a secondary dependency upon a single node with which node *x* originally interacted; this weakens the relative role of the new node in regulating node *x*. Essentially, this choice of methodology reflects the introduction of biological agents (e.g., synthetic signaling proteins [41]) that facilitate novel interactions that can drive system behavior in place of, or in conjunction with, the pre-existing interactions. While such an introduction, strictly speaking, increases the number of regulatory components in the system, we here choose to represent the agent only through its effect of modifying or creating an interaction between two pre-existing components of the system; this is a logical simplification because the introduced agent is designed explicitly to have no other effect on the system.

**Table 1:** Overview of the network repair algorithm

**#**	**INPUT**
1	A network, comprising update rules for all constituent nodes *x*_1_, …, *x*_*N*_
2	The state of every node *x*_*i*_ for every state of the attractor of interest, *a*_*s*_.
3	The damaged node *x*_*d*_ and the state to which it is to be forced.
4	*LCSS*, set to *True* if a limit cycle superset is to be considered and *False* otherwise.
#	**OUTPUT**
1	The nodes whose update rules must be modified to ensure the stability of the damaged attractor, *a*^*d*^.
2	the viable update rule modifications, for each of the nodes from (1).
	*or*
2	In the case of limit cycle repair failure, the cause of failure.
**#**	**ALGORITHM**
1	determine the damaged attractor *a*^*d*^.
2	**if***LCSS*: set *a*^*d*^ to be its superset.
	*determine the sensitive nodes:*
3	**for** every node *x*_*i*_ in every state *s* in *a*^*d*^:
4	update node *x*_*i*_ from state *s* with all other nodes held constant
5	**if***x*_*i*_ changes its state as a result, it is sensitive. Record initial and final states.
6	**if** any sensitive node has initial and final states [0,1] and [1,0]:
7	**return** "limit cycle repair failure, case 1"
	*determine all possible modifications for sensitive nodes:*
8	define an empty dictionary *R*
9	**for** every sensitive node *x*_*i*_ in every state *s* in *a*^*d*^:
10	**if** the next state of *x*_*i*_ must be OFF for repair:
11	record all combinations of nodes that obey each rule listed in col1 of Table 1.
12	**else:**
13	record all combinations of nodes that obey each rule listed in col2 of Table 1.
14	**for** every sensitive node *x*_*i*_:
15	set *R*[*x*_*i*_] = the intersection of the viable (rule, node) pairs across all states in *a*^*d*^
16	**if***R*[*x*_*i*_] is the empty set:
17	determine *R*[*x*_*i*_] when omitting states in *a*^*d*^ where node *x*_*i*_'s next state is equal to its current state
18	**if***R*[*x*_*i*_] is the empty set: **return** "limit cycle repair failure, case 2"
19	**else**: **return** "limit cycle repair failure, case 3"
20	**return***R*

The algorithm used to determine interaction modifications that stabilize a network attractor in response to node damage is presented in a concise pseudo-code format. A user-friendly implementation is provided as an extension to the Python software package BooleanNet [26].

**Table 2:** The logical update function modifications considered in this article

**ON to OFF corrections**	**OFF to ON corrections**
*f*_*x*_ = … AND *a*_*new*_	*f*_*x*_ = … OR *p*_*new*_
*f*_*x*_ = … AND NOT *p*_*new*_	*f*_*x*_ = … OR NOT *a*_*new*_
*f*_*x*_ = … AND (*a*_*orig*_ OR *a*_*new*_*)*	*f*_*x*_ = … OR (*p*_*orig*_ AND *p*_*new*_*)*
*f*_*x*_ = … AND (NOT *p*_*new*_ OR *a*_*orig*_)	*f*_*x*_ = … OR (NOT *a*_*new*_ AND *p*_*orig*_)
*f*_*x*_ = … AND (NOT *p*_*new*_ OR NOT *p*_*orig*_)	*f*_*x*_ = … OR (NOT *a*_*new*_ AND NOT *a*_*orig*_)
*f*_*x*_ = … AND (*a*_*new*_ OR NOT *p*_*orig*_)	*f*_*x*_ = … OR (*p*_*new*_ AND NOT *a*_*orig*_)

The rule modifications considered for a node *x*. Initial dots are placeholders for the original rule for the node, which could be, e.g., *f*_*x*_ = y. *p* indicates an arbitrary node that is present (ON) in the current state of the network, *a* indicates an arbitrary node that is absent (OFF) in the current state of the network. Subscripts indicate whether the node in question is a *new* regulator for this node (i.e., did not exist in the original update rule) or existed as an *original* regulator. The top two rules in either column are the simplest, but place the greatest regulatory weight on the new interaction.

As we shall show, in networks where no node is regulated by every undamaged node, this methodology and choice of interaction rule modifications can unambiguously fix any state to be a steady state of the network, including time-averaged representations of multi-state attractors. However, this approach is in general not sufficient to fix a selection of states to be a limit cycle of the network. When repairing a limit cycle, a single rule modification must accommodate all state transitions for each node; furthermore, such a modification must be found for all nodes that require repair at any transition in the limit cycle. Failures of this approach fall into three categories.

In the first category, a node requires both an ON to OFF correction and an OFF to ON correction within a limit cycle; this is uncorrectable while scaffolding on the existing update rule. We show one such example in Figure 2(c-d)). The two-state synchronous limit cycle shown with grey-bordered edges and grey arrows in Figure 2(d) translates to the pair of states targeted by the dashed edges when node C is knocked out; the desired repaired attractor is shown by the blue arrows with unfilled tips. However, forcing these states to form a limit cycle entails forcing node A to update to 1 (i.e., 01001 - > 10001) when it normally updates to 0 (i.e., 01001 - > 00011) and vice versa (i.e., we desire 10001 - >01001 but instead 10001 - > 11001).

In the second failure category, a node requires multiple corrections of a single type within a limit cycle, but no single rule modification suffices to make the repair in all cases. We show an example of this failure case in Figure 1(e-f). Figure 1(f) is similar to Figure 1(d), but here the desired repair is for node E to transition to 1 when it normally updates to 0 at both states of the desired limit cycle. Because node E's only regulator is the damaged node, we are restricted to the rules in the top two rows of Table 2. However, the potential new regulators for node E (nodes A, B, and C) oscillate in the two desired states (10101 and 01001), and therefore cannot bring about the desired transition for node E in both states when considering only modifications of the form listed in Table 2.

In the third category of limit cycle repair failure, a repair exists for all necessary corrections, but none preserve an original state transition that requires no modifications. In the example of Figure 2(g-h), the original attractor (10101 < − > 01101) translates into the desired pair of states (10101 and 00101) when node B is knocked out. However, in the damaged and unrepaired network, both node A and C fail to transition as desired from the state 00101. Node A is regulated by every node other than node E; node E must therefore fill the role of the new regulator of node A that takes it from the OFF to ON state. Each of the possible repairs, listed in rows 1, 3 and 6 of column 2 in Table 2, forces node A to update to 1 from the 00101 state; however, none of them ensures that A becomes inactive when updating from the 10101 state, and therefore the overall limit cycle repair is not possible.

More thorough interaction reformatting can, in general, alleviate these issues and allow for the repair of limit cycles. In addition, in cases where the dynamic fluctuations represented by a limit cycle are not requisite components of proper system function, the methodology presented here may be used to achieve an alternative stabilized network dynamics. We define the superset of a limit cycle as a single state wherein every node that is at least transiently active in the limit cycle is active, and all other nodes are inactive. For example, both limit cycles in Figure 1(b) have a superset of "1111", as all nodes are at least transiently active, the superset of the original limit cycle on Figure 2(b) is 11111, and the superset of the damaged limit cycle of Figure 2(b) is 11011. As discussed above, the network may be modified to ensure that the superset is a steady state, to preserve the original limit cycle's time-insensitive activation levels.

This choice of superset definition explicitly incorporates all nodes that are active to any extent in a time-averaged assessment of attractor dynamics. In some contexts, alternative definitions may be more appropriate; for instance, only nodes that are ON for a specified fraction of the limit cycle's states may be considered ON in the superset. Notably, the details of the process by which the states of a limit cycle are collapsed to a single representative state do not influence the ability of the methodology proposed here to fix that state to be a steady state of the modified network.

### Network repair

We now treat in detail the algorithm of Table 1, whose most complex step scales as *O*(*N*^2^) with N equivalent to the number of nodes in the network. A user-friendly implementation is also provided as an extension to the Python software package BooleanNet [26]. The first step (Table 1, line 1) is to determine the attractor *a*^*d*^ (i.e., the attractor to be stabilized), and, if desired, to collapse it to a single representative state (Table 1, line 2), as discussed in the previous section. The second step (Table 1, lines 3–5) is to identify the nodes that are deregulated within the attractor as a direct result of the network damage. Consider an arbitrary attractor composed of states *a*_1_, …, *a*_*n*_ (*n* ≥ 1). By definition, a node *x*_*i*_ that is not impacted by damage to node *x*_*j*_ will not change its state (for steady states) or usual progression of states (for limit cycles) as a direct result, i.e.

$$\begin{array}{l} f_{i}\left(x_{1}\left(t\right),\ldots ,x_{j-1}\left(t\right),0,x_{j+1}\left(t\right),\ldots ,x_{n}\left(t\right)\right)\\ \;=f_{i}\left(x_{1}\left(t\right),\ldots ,x_{j-1}\left(t\right),1,x_{j+1}\left(t\right),\ldots ,x_{N}\left(t\right)\right) \end{array}$$

for all *a*_*k*_, *k* = 1,…,*n*. That is, a node is *robust* to the network damage if the damage is not sufficient (with all other node states fixed) to cause it to change its state from any of the attractor states, and is otherwise *sensitive* to the damage. Sensitive nodes must have their update function *f*_*i*_ modified to become robust. Consider for example the desired attractor of Figure 1 (i.e. 1101): only node A is sensitive; it is regulated only by node C, the knockout of which is considered in Figure 1(d).

Importantly, because modifications to the node update functions are applied simultaneously to the damaged state(s) $a_{1}^{d},\ldots ,a_{n}^{d}$, the stability of every node may be individually considered under the assumption that all other nodes are stable. Moreover, only the regulatory targets of the damaged node need to be considered, as only they may suffer an immediate deregulation due to the network damage. Therefore, the computational complexity of this task scales as the product of the number of states in *a*^*d*^ and the number of regulatory targets of the damaged node; these are generally both quite small.

Limit cycle failures of the first type may be assessed at this point by recording all transitions (ON to OFF and OFF to ON) among the sensitive nodes (Table 1, lines 5–7). If no such failures are identified, the next task is to determine all viable interaction modifications that bring about node robustness. This consists of iteratively considering every sensitive node *x*_*i*_ in every state of the target attractor *a*^*d*^ (Table 1, lines 9–13). For every such combination, all other nodes are either ON or OFF and are either a regulator of *x*_*i*_ or not; these properties determine which nodes can be used with which rules in Table 2 to ensure the desired next state of node *x*_*i*_. As some of these rules consider node pairs as potential regulators, the complexity of this task scales as *O*(*N*^2^).

Steady state and limit cycle superset repair terminates at this point; the nodes and the viable node and rule combinations are returned. In the case of limit cycles, however, only the approaches that are applicable to all states in *a*^*d*^, for a given sensitive node, suffice (Table 1, lines 14–15). If there are no node and rule combinations that exist in all state transitions for one or more sensitive nodes, the method fails. Additional analysis may then be applied (of complexity *O*(*N*)) to differentiate between the above-discussed classes of failure (Table 1, lines 16–19). We apply this methodology to the example of Figure 1; one viable modification to the update rule for node A in response to the damage shown in Figure 1(d) is shown in Figure 1(g), and the resulting state transition networks for synchronous and asynchronous dynamic schemes are shown in Figure 1(h-i).

### Simulations to test the success of the method

**Figure 3:**
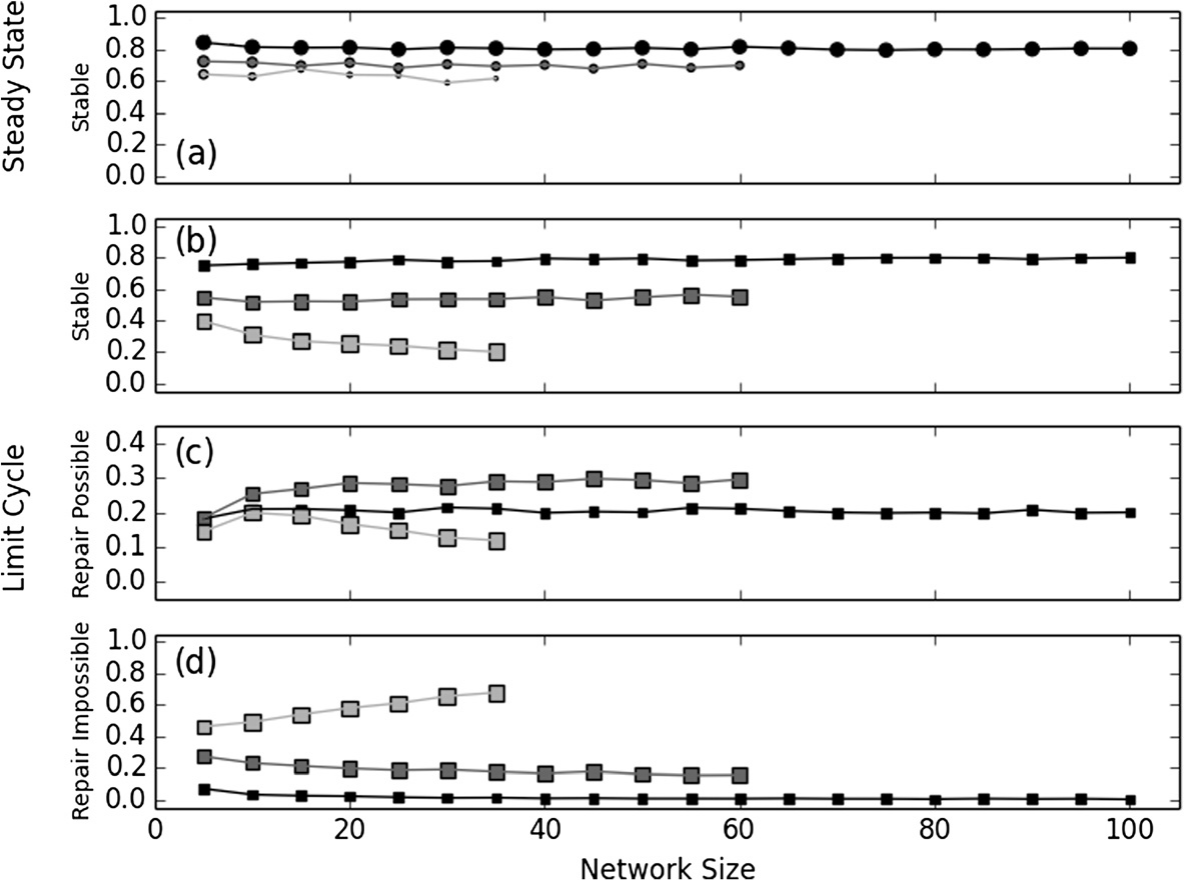
**Stability and repair frequency in random Boolean networks.** Synchronously updated random Boolean networks with *k* = 1, 2, and 3 regulators per node (black, dark gray, and light gray, respectively) are allowed to reach a steady state (circles) or a state that belongs to a limit cycle (squares), then are damaged by randomly selecting a node and fixing it to its opposite state. Symbol size corresponds to the fraction of observed steady states (panel **(a)**) vs. limit cycles (panels **(b)**-**(d)**) in 10,000 attractors selected from unique networks of a given size and value of *k*. **(a)** While steady states are less commonly observed for larger networks when *k* > 1, those that are observed are resilient to node knockout at a frequency that depends on *k* but not network size. All instances of steady states that are not stable may be repaired without ambiguity. **(b)** Observed limit cycles are stable with a frequency that decreases with *k*. For *k* < 3 the frequency of stable limit cycles does not depend on the network size, but it decreases as network size increases for *k* = 3. **(c)** Unstable limit cycles are repaired with decreasing frequency for increasing *k,* and with decreasing frequency as network size increases for *k* = 3. **(d)** Limit cycles that may not be repaired (see Methods) grow more frequent with increasing *k*. The values corresponding to black, dark gray, and light gray squares from panels **(b)**-**(d)** sum to 1.

**Figure 4:**
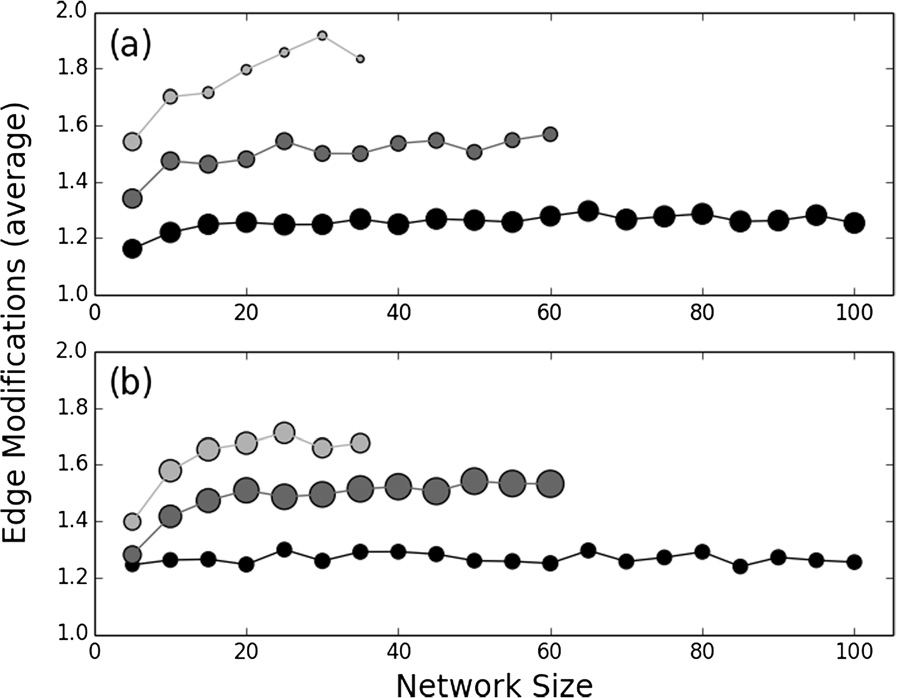
**Edge manipulations in random Boolean networks.** The average number of edge modifications required to stabilize **(a)** steady states and **(b)** non-truncated limit cycles for random Boolean networks with *k* = 1, 2, and 3 regulators per node (black, dark gray, and light gray, respectively). 10,000 networks were sampled for every combination of network size and *k*; symbol size corresponds to the distribution of these networks between panels **(a)** and **(b)**.

We first consider random Boolean networks with *N* nodes and *k* randomly assigned input nodes for every node (self-regulation is allowed). The future state of a node for each of the 2^*k*^ possible combinations of inputs is randomly assigned, without bias for either outcome. For every combination of *N* and *k* shown in Figures 3 and 4, we generated 10,000 networks. We then use a synchronous updating scheme to find an attractor of the network, and simulate damage by forcing a randomly selected node to be in its opposite state; that is, a transiently or permanently active node is forced to always be OFF, or a permanently inactive node is forced to always be ON.

In the case of steady states, if the damage-modified steady state is an attractor, the steady state is considered to be stable with respect to the damage. For limit cycles, if the damage-modified states of the limit cycle still constitute a limit cycle, or if they contain a smaller limit cycle (in cases of state merging), the limit cycle is considered to be stable with respect to the damage. For example, on Figure 2(b) state 11101 of the original limit cycle merges with state 11001 of the original limit cycle when node C is knocked out. Thus a limit cycle of five states would be considered stable with respect to the damage. However, the damaged limit cycle of four states on Figure 2(b) is not sufficient and needs repair. If the limit cycle is not stable sensitive nodes (i.e., those that fail to transition as in the pre-damage attractor) are identified.

For both the random Boolean networks and the biological case studies, all possible rule modifications (all possible combinations of nodes applied to rules listed in Table 2) are considered; in the case of random Boolean networks one rule is chosen at random for each sensitive node in each attractor. Once an update rule modification has been applied to all sensitive nodes, the stability of the attractor of the repaired network is evaluated. In cases where a limit cycle is unable to be repaired, the category of failure (as outlined above) is recorded.

The biological case studies involve straightforward application of the network repair methodology discussed here. Dynamics in the T-LGL case study are performed with the general asynchronous updating scheme wherein a randomly selected node is updated at each step. The update process in this scheme is a Markov chain; its attractors may be found by summarizing all possible state transitions in the transition matrix **T**, where **T**_*i,j*_ is the probability of state *i* updating to state *j* in a single update. Entries in every row of **T** sum to 1; rows with a single nonzero entry **T**_*i,i*_ correspond to steady state attractors. Complex attractors may be determined from **T** by determining the terminal strongly connected components of the corresponding transition graph.

Alternatively, because the probability of a state *i* updating to state *j* after *m* steps is given by $T_{i,j}^{m}$[42], evaluating $T_{i,j}^{m}$ for sufficiently large *m* reveals the relative probability of reaching every attractor from all possible source states. Specifically, any nonzero column *j* corresponds to a state that exists in an attractor, and the column entries indicate the relative frequency with which the system will be in the given terminal state *j* when initialized from source state *i*. The complexity of this procedure is *O*(*mn*^*x*^), with *x* = 2.38 for the standard Coppersmith-Winograd algorithm for multiplication of two *n* × *n* matrices [43]. Here, the transition matrices were computed through exhaustive simulation of all possible transitions from every network state. The attractors were then determined by computing the nonzero values (>10^−5^) with *m* > 10^3^.

## Results

The methodology outlined above may be applied to any Boolean network with logical update rules. We first show the analytical result that the methodology outlined here may be used to stabilize any single steady state or limit cycle superset of a wide class of networks. We then apply the methodology to (1) random Boolean networks and (2) two biological cases studies. In case (1), we show the ability of the method to repairing both steady states and synchronous limit cycles for a robust range of network parameters, and in case (2) we show that the method is able to quickly provide insight into possible methods for intervention in real networks.

### Stabilization of a single state

#### Theorem

Any state *σ* of a discrete time Boolean network may be modified to be a steady state of the network through application of the rule modifications listed in Table 2 if no node is regulated by every undamaged node in the network and if the future state of every node depends only on the current state of other nodes.

#### Proof

Consider the network to be in state *σ* at time *t*. Then all nodes *x*_*i*_ that obey *x*_*i*_(*t*) ≠ *x*_*i*_(*t* + *τ*_*i*_) are candidates for modification according to Table 2. For every such node *x*_*i*_, consider an arbitrary node *x*_*j*_ that does not regulate node *x*_*i*_ (*x*_*j*_ may be the same node as *x*_*i*_ in cases where the node does not self-regulate). The four modifications listed in the top two rows of Table 2 indicate a sufficient rule modification by adding *x*_*j*_ as a new regulator for each of the four combinations of states of nodes *x*_*i*_ and *x*_*j*_ at time *t*. The remaining modifications listed on Table 2 involve *x*_*j*_ in combination with existing regulators of *x*_*i*_.

In this article, the state *σ* may be viewed as a damaged steady state; the nodes that require interaction modification are those that are sensitive to the network damage. The ability for this approach to stabilize limit cycle supersets follows directly from the fact that the methodology is sufficient to stabilize *any* single state.

### Random Boolean networks

The density of connections in random Boolean networks has a strong influence on the frequency with which both steady state and limit cycle attractors are resilient to network damage (i.e., require no node rule modifications to preserve the attractor in its damage-modified state). Specifically, on average 81%, 70%, and 63% steady state attractors are resilient to damage for *k =* 1, 2, and 3, respectively (see Figure 3(a)) and on average 78%, 54%, and 27% of limit cycles are resilient to damage for *k =* 1, 2, and 3; see Figure 3(b). Of the steady state attractors that are not robust to damage, all (100%) were stabilized after application of the update rule modifications as described in the Methods section, i.e., by simultaneously modifying the update rules for all sensitive nodes, we unambiguously ensure that the damaged state becomes a steady state in the repaired network (that $a^{d}\to a_{s}^{d}$).

The rule modifications considered in this article are not able to always restore the complete dynamics of the damage-modified limit cycle (1%, 18%, and 54% failure rate for *k* = 1, 2, and 3, respectively; Figure 3(c) cf. Figure 3(d)). Most failures are categorized as case (3) discussed above: update rule modifications that correct undesirable transitions cannot also preserve desirable node state transitions within a limit cycle (accounting for 96%, 71%, and 58% of the failures, for *k* = 1, 2, and 3, respectively). Most of the remaining cases of failure fall under case (1) discussed above: a node requires both types of correction and may not be corrected with the rule modifications considered here. However, collapsing the limit cycle to its superset (see Methods) reduces the problem to that of a steady state of the network, and is therefore always successful.

The average number of edge modifications necessary to repair the network does not vary based on network size, for both steady state and limit cycle attractors (Figure 4). Furthermore, the average number of modifications required increases only slightly as the nodal in-degree increases. The upper bound on the number of edge modifications that are required in response to the deregulation of a single node is equal to the node's out-degree. The out-degree distribution of random Boolean networks is centered around the average out-degree value, which equals the fixed in-degree of each node. Nonetheless, the average number of edge manipulations necessary for network repair was well below the average out-degree of the networks for the *k* = 2,3 cases: steady states required an average of 1.50 and 1.77 edge modifications across all simulations, respectively, while repairable limit cycles required an average of 1.48 and 1.62, respectively. This suggests that in the context of sparse random networks, when network repair of a destabilized attractor is possible, it may be achieved through a minimal intervention, regardless of network size or edge density.

The number of interventions necessary for the superset-based repair of damaged limit cycles does depend on the size of the network. In a bias free random Boolean network, the probability of a node changing its state based on any input is *p* = .5. The number of nodes that change their state in a single step from a randomly selected state in a random Boolean network of size *N* is therefore *pN*, regardless of edge density. Since the state that corresponds to the superset of a limit cycle, with one node additionally damaged, may be as far away from a steady state as a randomly selected state, the expected number of required edge modifications is *pN*. However, limit cycles in real biological systems may be largely stable in that few nodes change their state over the period of the limit cycle [28]. This implies greater superset stability than in random networks; as such, the stabilization of limit cycle supersets in biological systems may require fewer interaction modifications than in the case of random networks.

### ABA induced closure of plant guard cells

Most plants regulate their uptake of carbon dioxide through stomata: microscopic pores that coat much of the epidermis of the plant. The aperture size of a stoma is regulated by a pair of guard cells, which contract or relax in response to environmental cues. While stomatal opening is required for the uptake of carbon dioxide, open stomata facilitate evaporative loss of water from the plant (i.e., transpiration). In drought conditions, plants therefore close their stomata, a process which is induced by the plant hormone abscisic acid (ABA) and involves protein-protein interactions, biochemical reactions and ion transport.

We consider the ABA signal transduction network constructed by Li et al. [31], and further studied in [44]. This network has a single attractor in the persistent presence of ABA, a steady state (fixed point) in which the node closure is in the ON state. All initial conditions that include the presence (ON state) of ABA converge to this closure steady state. In [31], the authors found that single knockouts of 10 of the 43 nodes of the network significantly impaired stomatal closure, as measured by the frequency with which closure stabilized in the ON state when sampling the space of all initial configurations of the non-source and non-sink nodes. In 3 of these cases (knockout of the nodes Depolar, AnionEM, or Actin) closure was completely inhibited, while in the remaining 7 (knockout of the nodes PLD, PA, SphK, S1P, GPA1, KOUT, or pH_c_), closure was partially inhibited. All other single-node knockouts affected at most the number of time steps required to achieve closure.

We therefore consider repairing this network in response to the knockout of the 10 key regulatory nodes. In [31], dynamics are simulated on the network via the random order asynchronous (ROA) scheme (see Methods). We find that the repair methodology introduced in this article successfully repairs damage applied to the closure = ON steady state attractor of the ABA network. In this procedure, we begin from the damaged steady state wherein a particular node is fixed in its opposite state and identify rule modifications that prevent the propagation of the damage. Interestingly, the knockout of KOUT (which represents K^+^ efflux through the plasma membrane) does not otherwise alter the stability of the healthy attractor of the network and therefore requires no network modification. In [31], the authors found that after knocking out KOUT and randomly sampling all possible initial conditions, closure = ON states were found with decreased frequency. Taken together with the result of this analysis, we find that the original attractor survives a KOUT knockout, although it becomes difficult to reach from other states. This is in contrast to the other considered cases of network damage, which lead to the further collapse of the original attractor unless network modification occurs. Indeed, all other knockouts required modifying the update rules for 1, 2, or 3 nodes; the search algorithm identified roughly between 80 and 300 possible rule modifications for each of these nodes.

We consider in detail damage to heterotrimeric G protein α subunit 1 (abbreviated as GPA1 in [31]), and determine that 16% of the possible repairs introduce one or more undesired secondary attractors wherein closure is OFF. This demonstrates the point that analysis of alternate attractors is an important consideration when characterizing network repairs that, in themselves, ensure only the stability of an attractor, and not the extent to which it is reachable.

**Figure 5:**
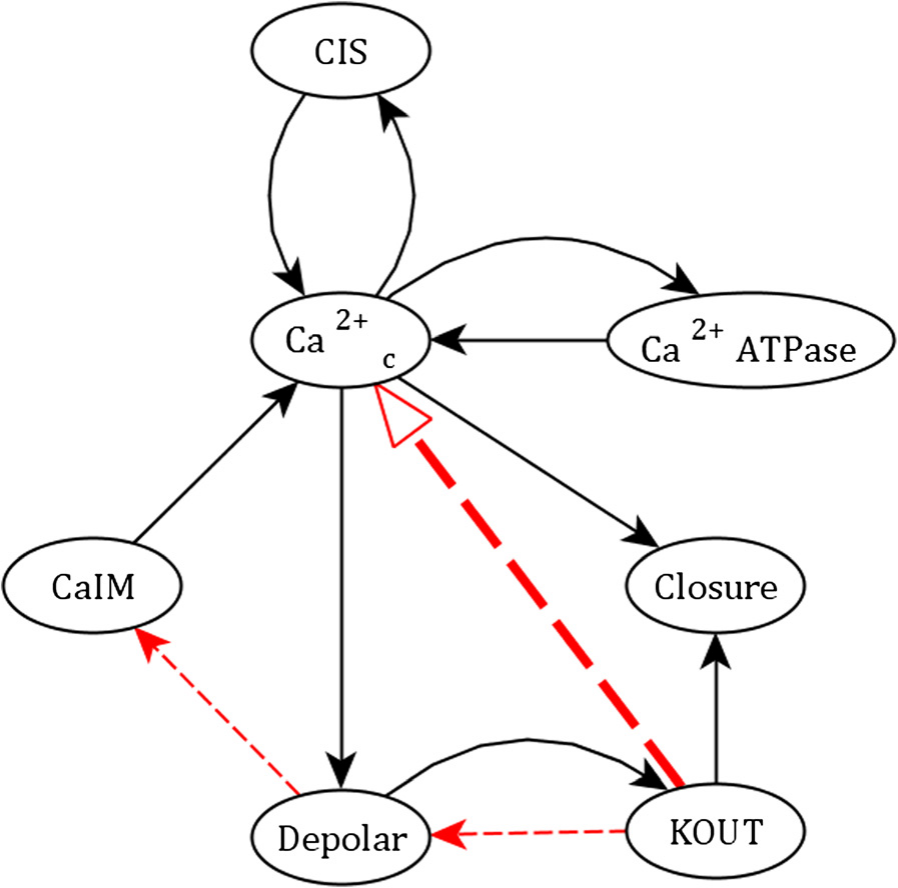
**The modified reduced ABA signaling network.** The 7 node reduced network of [44], adapted from the full model of [31]. Black arrows represent activation, and red dashed arrows represent inhibition. The bold red arrow from KOUT to Ca^2+^_c_ is a possible addition to the network that preserves the limit cycle of the unmodified and undamaged network in response to the constitutive loss of CaIM. The update rules and limit cycle for this network are provided in Tables 3 and 4, respectively. The full names corresponding to the abbreviated node labels are (see [31]): CIS, Ca^2+^_c_ influx to the cytosol from intracellular stores; Ca^2+^_c_, cytosolic Ca^2+^ increase; Ca^2+^ ATPase, Ca^2+^ ATPases and Ca^2+^/H^+^ antiporters responsible for Ca^2+^ efflux from the cytosol; CaIM, Ca^2+^ influx across the plasma membrane; Depolar, plasma membrane depolarization; KOUT, K^+^ efflux through slowly activating outwardly-rectifying K^+^ channels at the plasma membrane.

We also consider a 7-node reduced version of the ABA induced closure network obtained in [44] (Figure 5, Table 3). The sole attractor of this network in the synchronous updating scheme is a 5-state limit cycle (Table 4); asynchronous schemes yield a large complex attractor [44]. We iteratively knock out each of the 7 nodes of the reduced model. In the case of two nodes, CIS and Closure, the surviving portion of the limit cycle is stable (see Methods). For the remaining five knockouts, the network repair methodology succeeds in repairing the limit cycle in two cases (CA^2+^ATPase, CaIM), or fails under the second (Depolar) or third classification (KOUT, Ca^2+^_c_), as discussed in the Methods. As is always the case, the methodology proposed here is able to collapse the limit cycle into its superset, the steady state wherein all undamaged nodes transiently present in the undamaged limit cycle are present. We highlight in particular the successful repair of the limit cycle when CaIM is knocked out. As shown in Figure 5, CaIM regulates Ca^2+^_c_, which depends critically upon CaIM for 2 of the 5 state transitions in the limit cycle. KOUT is OFF in both of the relevant states, but ON in all others; appending "*OR NOT KOUT*" to the update rule for Ca^2+^_c_ therefore successfully preserves its affected state transitions without influencing any others (Table 3). As in the full model, we consider the effects of the repair on the topology of the state transition network. This repair introduces no alternative attractors; i.e., the system will always dynamically evolve to the repaired attractor.

**Table 3:** The update rules for the reduced ABA network model

*f*_*CIS*_	=	Ca^2+^_c_
*f*_*Ca2+ATPase*_	=	Ca^2+^_c_
*f*_*Ca2+c*_	=	***(*** (CaIM or CIS) and (not CA^2+^ATPase) ***) or not KOUT***
*f*_*Depolar*_	=	(not KOUT) or Ca^2+^_c_
*f*_*CaIM*_	=	not Depolar
*f*_*KOUT*_	=	Depolar
*f*_*Closure*_	=	KOUT and Ca^2+^_c_

The ABA model is shown in Figure 5. Bold, italicized text represents a possible rule modification that preserves the network's limit cycle in response to the constitutive knockout of CaIM. Node abbreviations are given in the caption to Figure 5.

**Table 4:** The attractor of the reduced ABA network model

**CIS**	**Ca**^**2+**^_**c**_	**CA**^**2+**^**ATPase**	**CaIM**	**Closure**	**Depolar**	**KOUT**	
1	0	1	0	1	1	1	
0	0	0	0	0	0	1	
0	0	0	1	0	0	0	***Critical***
0	1	0	1	0	1	0	***Critical***
1	1	1	0	0	1	1	

The ABA model is shown in Figure 5. The states labeled as critical in bold, italic text are those where deregulation of CaIM will lead to the deregulation of Ca^2+^_c_ in the following time step (when updating synchronously). KOUT has one state during these critical time steps, and the other state in all other cases, indicating that it can compensate for the loss of CaIM through the rule modification to Ca^2+^_c_ shown in Table 3, without deregulating Ca^2+^_c_ in other time steps.

### T-LGL leukemia

T cell large granular lymphocyte (T-LGL) leukemia is characterized by unregulated proliferation of cytotoxic T cells [32,34]; this expansion continues unchecked due to the deregulation (malfunctioning) of the natural process of activation induced cell death (apoptosis) [45]. No curative therapy exists for T-LGL leukemia; understanding the signaling pathways that deregulate apoptosis is a prerequisite to the development of therapeutic treatments that restore the natural process of cell death.

**Figure 6:**
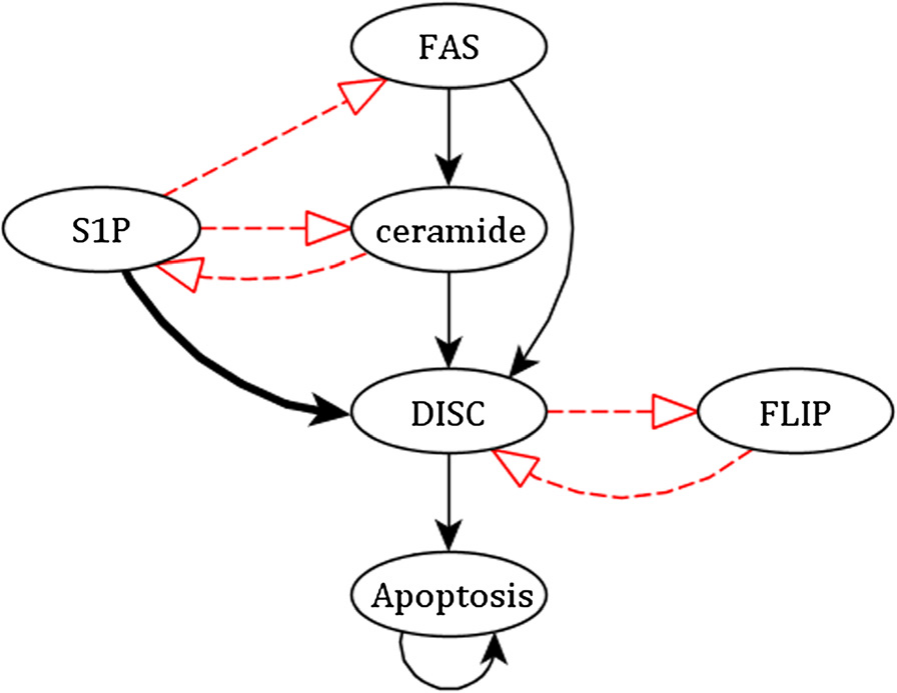
**The modified reduced T-LGL network.** The 6 node reduced network is adapted from [34]. Red dashed lines indicate negative regulation while solid black lines indicate positive regulation. The bold line represents a possible modification that eliminates the Apoptosis = OFF (cancerous) steady state. The full names corresponding to the abbreviated node labels are (see [34]): S1P, Sphingosine-1-phosphate; DISC, death inducing signaling complex; FLIP, CASP8 and FADD-like apoptosis regulator.

We consider an asynchronous Boolean network model of T cell survival signaling in the context of T-LGL leukemia [32,34]. In [34], network reduction techniques were applied to the full 60 node network to reduce it to a 6 node network that captures the salient behavior of the network (Figure 6, Table 5). This model employs a general asynchronous update (GA) scheme; because the GA scheme is not deterministic, the same initial condition may lead to multiple attractors depending on the specific order of state transitions. Indeed, the authors found that of the 64 dynamic states of the 6-node reduced network, 25 evolve to either a healthy (apoptosis = ON) fixed state or to a T-LGL (apoptosis = OFF) fixed point, depending on the trajectory of the asynchronous update process. 3 states exclusively lead to the T-LGL fixed point, while the remaining 36 exclusively lead to the apoptosis steady state.

As in the case of the ROA update scheme discussed in the previous case study, the edge modification methodology presented in this article may be applied directly to a GA update scheme. Indeed, the GA scheme is identical to the ROA scheme with the exception of that the GA scheme does not require every node to be updated *m* times before any node is updated *m* + 1 times; as steady states are consistent across all dynamic update schemes, this nuance does not alter the effect of edge modifications in the context of preserving steady states.

Aside from the trivial case of forcing apoptosis itself to be OFF, the existence of the apoptosis = ON steady state is robust to all single node perturbations, because apoptosis self-regulates once active (n.b. forcing apoptosis to be OFF has limited biological meaning, as apoptosis is an outcome of cellular signaling, rather than a molecular entity). Rather than investigating the preservation of the apoptosis = ON steady state, therefore, we choose to investigate the elimination of the diseased state; i.e., instead of adjusting network update rules to *preserve* a network attractor, we adjust the update rules to *remove* an attractor.

Apoptosis is initially activated by DISC; we choose to modify its update rule in such a way as to allow its activation when the system is in the T-LGL state (where S1P = FLIP = ON and all other nodes are OFF). To account for the fact that the node rules in this network are designed to reflect the deactivation of all signaling components upon cell death, we consider rule modifications within the context of an overarching OFF signal from apoptosis. A rule modification that obeys the criteria outlined in this article is an additional dependency on S1P, such that the update rule for DISC changes from *f*_*DISC*_ *= (ceramide OR (FAS AND NOT FLIP)) AND NOT apoptosis* to *f*_*DISC*_ *=* ***(*** *(ceramide OR (FAS AND NOT FLIP))* ***OR S1P)*** *AND NOT apoptosis* (emphasis added for clarity; see Figure 6, Table 5). S1P acts as an inhibitor of FAS and ceramide, which contribute to the activation of DISC in the original network; bypassing this cascade and setting S1P to directly activate DISC ensures that DISC will always be activated in an otherwise unperturbed network.

**Table 5:** The update rules for the reduced T-LGL network model

*f*_*S1P*_	=	not (Ceramide or Apoptosis)
*f*_*FLIP*_	=	not (DISC or Apoptosis)
*f*_*FAS*_	=	Not (S1P or Apoptosis)
*f*_*Ceramide*_	=	FAS and not (S1P or Apoptosis)
*f*_*DISC*_	=	***(*** (Ceramide or (FAS and not FLIP)) ***or S1P)*** and not Apoptosis
*f*_*Apoptosis*_	=	DISC or Apoptosis

The T-LGL model is shown in Figure 6. Bold, italicized text represents a possible rule modification that eliminates the network's T-LGL (cancerous) steady state. Node abbreviations are given in the caption to Figure 6.

Indeed, we find that the network modified in this way has only one attractor, identical to the apoptosis = ON attractor of the original network; the entire state space is in the basin of attraction for this attractor. We note that this modification has a similar effect as the knockout of S1P, which also removes the T-LGL steady state [34]. While the outcomes are similar, this alternate approach highlights a different avenue for the development of curative therapies.

## Discussion

The deregulation of signal transduction networks, which can be brought about by the over- or under-expression of regulatory components, can lead to abnormal outcomes and ultimately to disease. When investigating network vulnerability, most studies identify nodes whose destabilization leads to drastically altered topological features or dynamical behavior in the network. The question of mitigating the effect of network damage, however, has received less attention.

From a dynamical systems perspective, the solution is clear: if two attractors are present, one desirable and the other not (as in the case of the T-LGL case study), modifying the expression levels of regulatory components to match the desirable attractor or a state in its basin of attraction suffices [39]. Alternatively, when considering damage in the form of node over- or under-expression, reversing the damage and returning the network to its original state clearly obviates the need for additional repair.

However, these approaches are often unrealistic in a practical sense. One alternative approach involves compensating for network damage by fixing the state of one or more initially unaffected nodes; this is possible in signal transduction networks, e.g. through gene manipulations, constitutive activation, or pharmacological interventions (see e.g. [46,47]). However, these modifications have an effect on every signaling component with which the targeted component interacts; in many cases, the deleterious effects of an intervention supersedes the intended benefit. An alternative approach, considered here, is modification not of node expression levels but rather of node interactions. The secondary effects are minimized, as only the chosen node (the target of the modified interaction) is directly affected. The empirical implementation of such a modification is highly dependent on context; for instance, in some cases synthetic signaling proteins could facilitate deliberate rewiring of signaling networks [41].

Clearly, directly inducing widespread modifications to expression levels of many regulatory components, or of the interactions between many such components, can be problematic. Minimization techniques are therefore of considerable interest. A strength of the approach introduced here is that it mitigates the effect of network damage at its source, in that it identifies a minimal set of network interaction modifications that preserves the stability of a network attractor (or, alternatively, eliminates an undesirable attractor). We consider modifications to the update rules for only those nodes that initially deviate from a desired attractor, and in so doing stymie the failure cascade that can otherwise send the system to a drastically different attractor. While many complex rule modifications are conceivable, we here focus on additions to the existing update rules (Table 2), using a novel regulator for the target node, and in some cases an existing regulator in an auxiliary role.

We explore the limits of this methodology, as related to network complexity (size and number of node inputs) in the context of synchronously updated random Boolean networks. We confirm our analytical result that the method ensures that the damage-modified steady states remain steady states with 100% frequency. A key factor here is the relative timing of system interactions; our methodology may be applied in a straightforward way to various deterministic [48,49] and stochastic [50-52] timing schemes. Where the method breaks down in the case of limit cycles, we note that (1) more substantial modifications to update rules may suffice to repair the damaged network and (2) the superset equivalent of a limit cycle (i.e., the state where all nodes that are at least transiently ON in the limit cycle are ON, and others are OFF) may be modified to be a steady state using the methodology outlined for steady states; clearly, however, this destroys the dynamics of a limit cycle and is not an appropriate strategy in situations where cyclic dynamics are an important property of the attractor. Indeed, network modifications must be considered in the context of the entire system in which the network is embedded, not only in the interests of practicality of implementation but also in order to understand the potential ramifications of manipulating the network. Indeed, we stress that the method, as presented here, considers the preservation of a single attractor. When the stabilization of multiple attractors is of interest, each may be considered separately and joint solutions may be identified from the set intersection of the individual solutions. Furthermore, while this methodology effectively identifies avenues for network repair, it does not explicitly consider the impacts of the repair on the state transition network: proposed repairs must be carefully filtered for the possible introduction of undesired attractors or other undesired effects on the system's dynamics.

The utility of this approach has been demonstrated in two biological case studies. In the case of drought-induced signaling in plants, we identify regulatory modifications that stand to protect the signaling network of the plants against the deregulation of key nodes. Resilient signaling in response to drought is an important consideration for agriculture; moreover, these insights provide testable hypotheses that may lead to further insight into the functioning of this complex regulatory network. In the T-LGL leukemia case study, we demonstrate alternative approaches for the elimination of the diseased T-LGL cell state; such an approach may lead to therapies for the removal of T-LGL cells *in vivo*.

## Conclusions

We have shown that in the context of random Boolean networks, successful network repair can be achieved with few edge modifications, with minimal dependence on the size or interaction density of the network. The upper bound on the number of edge modifications that are required in response to the deregulation of a single node is equal to the node's out-degree. In contrast to the peaked out-degree distribution of random Boolean networks, large-scale biological networks have long-tailed out-degree distributions [53,54]. Clearly, the loss of a high-degree node may require many compensatory edge modifications. Nonetheless, given the low average out-degree of many intracellular signaling and regulatory networks [9,31,32,53-57], it seems that network engineering via interaction manipulations may, in many cases, require few manipulations to mitigate network damage -- in addition to the aforementioned benefit of increased specificity relative to modifications to node expression levels.

We have considered two case studies of signal transduction networks, and have shown that our methods are successful in both cases and for a variety of dynamic update schemes. While limit cycles are dependent upon the choice of updating scheme, steady state attractors are uniform across dynamic schemes; their stabilization, therefore, requires comparatively less information concerning system dynamics. Indeed, the methodology introduced in this article is well-suited for any system modeled by Boolean networks with logical update rules. We provide an implementation of the analytical tools described in this article as an extension to the freely available software package BooleanNet [26].

The approach outlined in this article represents a first consideration of the modification of regulatory interactions with the intent of network control. Further theoretical work is necessary to facilitate meaningful advances *in vitro* and *in vivo*, as a number of caveats apply to the methodology discussed here. An important consideration when examining modifications to regulatory interactions is their impact on the state transition network (see e.g. Figure 1): many biological systems respond dynamically to exterior stimuli, and are characterized in part by the ability to transition between dynamic attractors. In such instances, not only must the stability of an attractor be maintained, but the ability of the system to properly interpret exterior stimuli must also be considered. In addition, further minimization of the number of intervention targets should be possible. Nodes that are deregulated by network damage may not have a critical impact on other nodes (or, notably, on the output nodes of signal transduction networks, such as cell death), or may only regulate other nodes that are also immediately deregulated by the initial network damage. Depending on the goal of network influence, therefore, not all deregulated nodes are necessarily targets for intervention. Effective prioritization of node intervention will enhance the utility of the methodology outlined in this article. In addition, the preemptive network modification considered here is clearly not practical in all cases; adaptation of the methodology to the control of networks that have already experienced first deviations will facilitate the analysis of network control in broader contexts. Finally, computational optimization may be necessary for very large networks; one such approach may be to identify viable interacting partners based on limit cycle synchrony (see e.g. Table 4) prior to the combinatorial evaluation of node pairs for the complex rule modifications given in Table 2. Approaches that take these considerations into account may be compared to the approach outlined here, and in this sense this work will serve as an effective benchmark for future studies that consider modifications to regulatory interactions.

## Competing interests

The authors declare that they have no competing interests.

## Authors' contributions

CC designed the study, created the software, performed the analysis, and drafted the manuscript. RA designed the study and drafted the manuscript. Both authors read and approved the final manuscript.
